# A Proteomic Analysis Provides Novel Insights into the Stress Responses of *Caenorhabditis elegans* towards Nematicidal Cry6A Toxin from *Bacillus thuringiensis*

**DOI:** 10.1038/s41598-017-14428-3

**Published:** 2017-10-26

**Authors:** Bing Wang, Haiwen Wang, Jing Xiong, Qiaoni Zhou, Huan Wu, Liqiu Xia, Lin Li, Ziquan Yu

**Affiliations:** 1 State Key Laboratory of Developmental Biology of Freshwater Fish, College of Life Science, Hunan Normal University, Changsha, 410081 P.R. China; 2 State Key Laboratory of Agricultural Microbiology, College of Life Science and Technology, Huazhong Agricultural University, Wuhan, 430070 P.R. China

## Abstract

Cry6A represents a novel family of nematicidal crystal proteins from *Bacillus thuringiensis*. It has distinctive architecture as well as mechanism of action from Cry5B, a highly focused family of nematicidal crystal proteins, and even from other insecticidal crystal proteins containing the conserved three-domain. However, how nematode defends against Cry6A toxin remains obscure. In this study, the global defense pattern of *Caenorhabditis elegans* against Cry6Aa2 toxin was investigated by proteomic analysis. In response to Cry6Aa2, 12 proteins with significantly altered abundances were observed from worms, participating in innate immune defense, insulin-like receptor (ILR) signaling pathway, energy metabolism, and muscle assembly. The differentially expressed proteins (DEPs) functioning in diverse biological processes suggest that a variety of defense responses participate in the stress responses of *C*. *elegans* to Cry6Aa2. The functional verifications of DEPs suggest that ILR signaling pathway, DIM-1, galectin LEC-6 all are the factors of defense responses to Cry6Aa2. Moreover, Cry6Aa2 also involves in accelerating the metabolic energy production which fulfills the energy demand for the immune responses. In brief, our findings illustrate the global pattern of defense responses of nematode against Cry6A for the first time, and provide a novel insight into the mechanism through which worms respond to Cry6A.

## Introduction

*Bacilllus thuringiensis* (Bt), a Gram-positive ubiquitious soil bacterium, belongs to *Bacillus cereus* group^1^. The most striking characteristic of Bt strains is that they produce insecticidal crystal proteins during the sporulation phase^2^. Crystal proteins are highly toxic against different insect orders such as Lepidoptera, Diptera, Coleoptera, Hymenoptera, as well as to nematode^2^. Because of no adverse effect on human and animal health, crystal proteins are thus considered environmentally safe alternatives to chemical pesticides, and they are also genetically engineered into crops to provide constant protection^3^.

The widely accepted mode of action of the crystal protein is the classical pore-formation model, a complex mechanism involving multiple and sequential binding interactions with specific protein receptors located in the microvilli of mid-gut epithelial cell of insect^4^. Followed ingestion, the crystal proteins are activated by the gut protease of the susceptible insect, and then bind to the first receptor and the second receptor in turn. Upon the interaction with receptors, the monomeric toxin forms oliglmer, then inserts into the membrane, and generates toxin pores in the mid-gut cell membrane, eventually causes swelling, lysis of cell and death of insect^3–6^. Whereas, an alternative model of action of the crystal protein proposed that crystal protein toxin activates an Mg^2+^-dependent adenylyl cyclase protein kinase A signaling pathway by interacting with receptor in insect^7,8^. However, the signaling pathway has only been proven *in vitro* in insect cell line. Moreover, no subsequent experimental evidence supports this mode of action *in vivo*.

To date, among the diverse crystal proteins produced by Bt, several families of crystal proteins were observed to be toxic to nematode, such as Cry5, Cry6, Cry 12, Cry14, Cry21, and Cry55^9,10^. Cry5B and Cry6A represent two distinct nematicidal crystal protein families, and share less similarity at the level of their primary sequences and the structures^11^. Cry5B exhibits a similar structure with other insecticidal crystal proteins containing the conserved three-domain (3-d) architecture responsible for pore-forming^12^. However, Cry6A does not possess the typical 3-d architecture, and exhibits a distinct structure with Cry5B and other insecticidal crystal proteins^11^. Cry6A shows structural homology to hemolysin E from *Escherichia coli* and two *B*. *cereus* toxins: the hemolytic toxin HblB and the NheA component of the non-hemolytic toxin (pfam05791)^13^. Based on the architecture, Cry6A proteins are grouped into the alpha helical pore-forming toxins. This structure is not previously recognized among the crystal proteins of Bt, and represents a new paradigm for crystal proteins^13^.

The altered structures of Cry5B and Cry6A proteins from each other are responsible for the difference in mode of action in *Caenorhabditis elegans*, the model nematode. Previous studies have revealed that the mode of action of Cry5B is almost similar to pore-forming mechanism of other 3-d crystal protein toxins, but the only difference is, it utilizes the invertebrate-specific glycolipid as receptor^14–16^. Whereas, Cry6Aa toxin triggers the Ca^2+^-dependent calpain-cathepsin necrosis signal pathway in *C*. *elegans*, which is mediated by aspartic protease (ASP-1)^17^. This is a novel action mechanism, and is distinct from all reported modes of action of crystal proteins from Bt.

Upon exposure to harmful toxins or pathogens, nematode utilizes various defense responses such as behavioral defenses^18^, innate immune responses^19^ to protect itself from intoxication or infection. Previous studies have confirmed that *C*. *elegans* takes various innate immune responses to protect itself when intoxicated by Cry5B, such as activating the insulin-like receptor (ILR) signaling pathway^20^, p38 mitogen-activated protein kinase (MAPK) and c-Jun N-terminal-like kinase (JNK)^21^ pathway; releasing protest-type lysozymes^22^, glycolipid-binding galectin^23^; triggering unfolded protein response^24^, hypoxic response pathway^25^. Among these cellular defenses, p38 and JNK MAPKs form a core defense network, whereas, JNK MAPK is a key central regulator of Cry5B induced transcriptional and functional responses^26^. *C*. *elegans* could utilize all these innate immune responses to protect itself from the diverse range of pathogens and harmful toxins of its natural habitat. Therefore, all these defense responses are crucial for nematode survival under natural condition.

Focusing on the fact of distinct architecture and mode of action of Cry6A from Cry5B and even other insecticidal crystal proteins, we propose that the nematode defense response towards Cry6A is probably different than towards Cry5B. Although the defense responses of nematode against Cry5B have been clearly elucidated in previous studies, the mechanism of how nematode defends against Cry6A is still obscure. To this end, the global defense pattern of nematode against Cry6A was investigated by proteomic analysis in the current study. To our knowledge, this is the first time that the global pattern of nematode defense responses against Cry6A toxin was revealed. Our result confirmed that *C*. *elegans* could protect itself via diverse defense responses when challenged by Cry6A toxin. Moreover, this research demonstrated the divergence of nematode defense response to Cry6A and Cry5B, and provided novel insight on understanding the complicacy of nematode stress responses to diverse range of stimulators.

## Results

### Profiles of differentially expressed proteins in worms under exposure to Cry6Aa2 toxin

**Figure 1:**
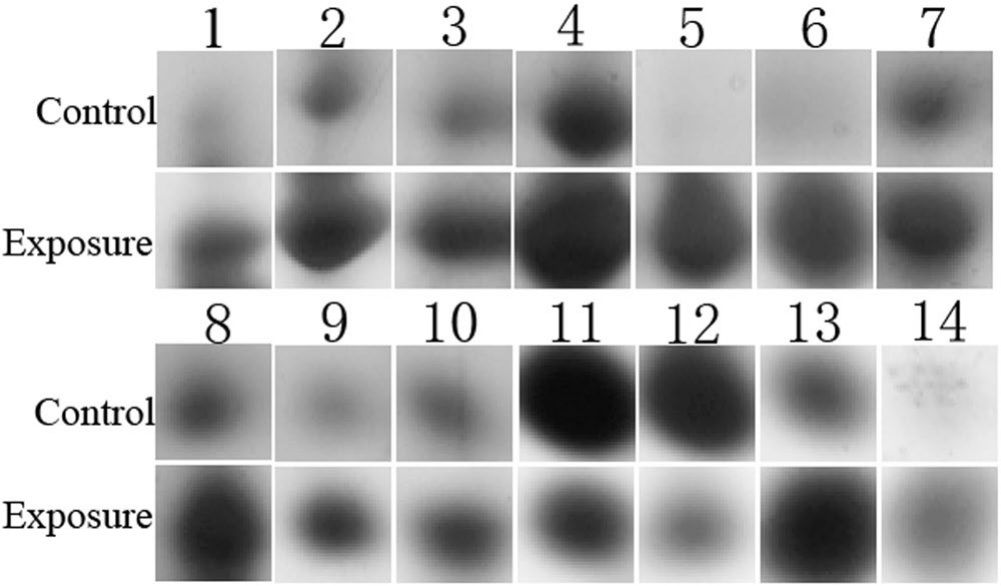
Differential analysis with zoomed images of a gel area from the N2 worms intoxicated by Cry6Aa2 toxin (Exposure) and from a comparable area of gel showing protein patterns from control worms unexposed to Cry6Aa2 toxin (Control).

To elucidate the changes of protein abundance in response to Cry6Aa2 toxin, 2-DE (two-dimension gel electrophoresis) was performed to compare the protein profiles of worms between exposure to Cry6Aa2 toxin and unexposure. To get accurate and credible data, all experiments were performed in triplicates. After Coomassie Brilliant Blue G-250 staining, more than 500 clear and reproducible protein spots were detected on each gel (Fig. S1). The comparative analysis of 2-DE maps of control and intoxicated worms was performed by PDQuest 8.0.1 software. A protein spot was considered differentially expressed when the protein had a fold change of more than two and a *P*-value less than 0.05. Based on the two criteria, a total of 14 differentially expressed protein (DEP) spots were observed with high confidence (*P* < 0.05) (Fig. 1). Among these DEPs, 12 of which were up-regulated and two were down-regulated by Cry6Aa2 intoxication (Table 1).

**Table 1:** Identification of the proteins differentially expressed in *C*. *elegans* when exposed to Cry6Aa2 toxin.

Spot no.	Protein name	Accession no.	Organism	Protein score	MW (kDa)/pI	Number of peptide sequences	Fold change (*P* < 0.05)^a^	Protein description
1	HSP-6	gi|17562024	*C*. *elegans*	155	71.1/5.89	7	3.46	Heat shock 70 kDa protein F, protein folding/response to stress
2	HSP-6	gi|17562024	*C*. *elegans*	493	71.1/5.89	17	5.24	Heat shock 70 kDa protein F, protein folding/response to stress
3	ENOL-1, isoform b	gi|32563855	*C*. *elegans*	66	36.5/5.47	4	4.23	Enolase, glycolysis
4	ENOL-1, isoform a	gi|17536383	*C*. *elegans*	728	46.8/5.56	19	2.23	Enolase, glycolysis
5	UCR-1	gi|17553678	*C*. *elegans*	599	51.7/6.07	21	6.76	Cytochrome b-c1 complex subunit 1, oxidation-reduction process/proteolysis
6	F46H5.3, isoform b	gi|32566409	*C*. *elegans*	227	40.4/6.17	13	4.78	Arginine kinase F46H5.3, kinase activity
7	DIM-1, isoform b	gi|351050470	*C*. *elegans*	137	35.8/5.17	11	2.38	Disorganized muscle protein 1, stabilizes the attachment of the myofilament lattice to the muscle cell membrane^34^
8	PDHB-1	gi|17538422	*C*. *elegans*	242	38.3/5.67	4	2.92	Pyruvate dehydrogenase E1 component subunit beta, glycolysis
9	F52H3.7b	gi|25154078	*C*. *elegans*	241	31.3/6.19	6	2.41	Galectin, LEC-2
10	HSP-25, isoform c	gi|71982762	*C*. *elegans*	108	12.4/5.89	3	2.13	Protein HSP-25, isoform c, response to stress
11	PUD-2.1	gi|373219676	*C*. *elegans*	227	17.4/6.04	6	-2.54	Up-regulated in *daf-2* ^28^
12	PUD-2.2	gi|17559735	*C*. *elegans*	123	17.4/6.04	4	-3.14	Up-regulated in *daf-2* ^28^
13	Y55B1AR.1	gi|17556226	*C*. *elegans*	136	15.98/6.17	3	2.62	Galectin, LEC-6
14	HSP-12.2	gi|17553934	*C*. *elegans*	105	12.31/6.28	4	12.37	Heat shock protein HSP-12.2, response to stress

^a^Numbers indicate fold-changes for up-regulated and down-regulated (−) expressions.

The 14 DEP spots were excised from the 2-DE gel and submitted to MALDI-TOF/TOF-MS for analysis. All the 14 proteins were identified with database searching. Based on the biological function, these DEPs were classified into the following functional categories (Table 1): energy metabolism (spot 3, 4, 5, 6, 8), heat-shock protein (HSP) (spot 1, 2, 10, 14), insulin-like receptor (ILR) signaling pathway (spot 11, 12), muscle assembly (spot 7), and galectin (spot 9, 13). Interestingly, spot 1 and spot 2 were all identified as heat shock 70 kDa protein F; spot 11 and spot 12 were identified as PUD (proteins up-regulated in long-lived *d* *af-2* mutant)-2.1 and PUD-2.2, while PUD-2.1 and PUD-2.2 are actually the same protein PUD-2^27^. In total, only 12 DEPs were actually observed in this study.

### Transcriptional analysis of the genes encoding DEPs

**Figure 2:**
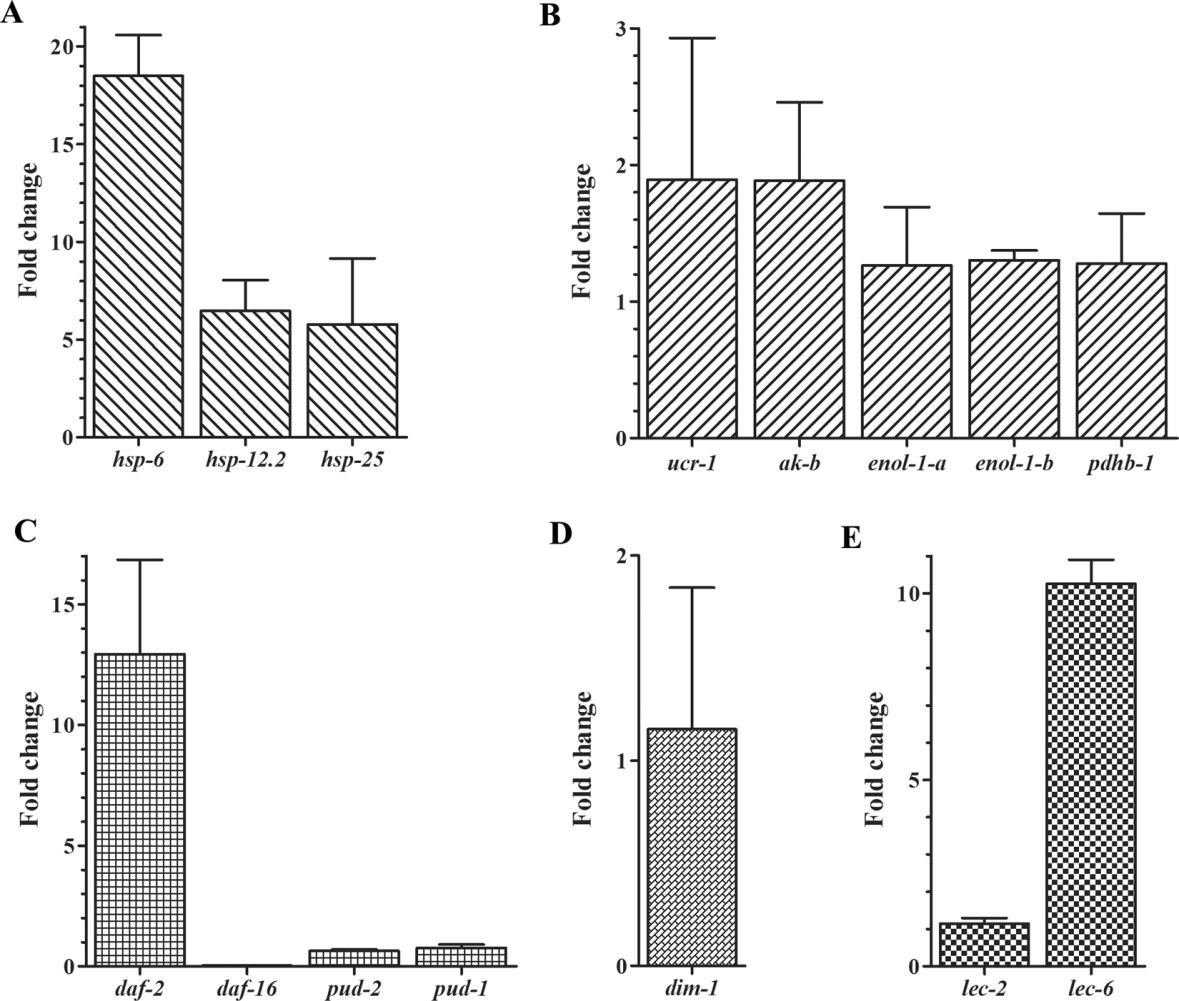
Relative expressions at mRNA level of genes from N2 worms intoxicated by Cry6Aa2 toxin. (**A**) genes encoding heat-shock proteins; (**B**) genes related to energy production metabolism; (**C**) genes involved in the insulin-like receptor signaling pathway; (**D**) *dim-1* gene; (**E**) genes encoding galectins. The fold change of mRNA is expressed as the ration of the mRNA level of worms exposed to Cry6A toxin/the mRNA level of control worms. Data are shown as the mean ±  SD.

From worms intoxicated by Cry6Aa2 toxin, 12 DEPs were observed by 2-DE as discussed earlier. In order to further verify the changed expression in transcriptional level and evaluate the correlation between mRNA level and protein level, the mRNA levels of genes encoding DEPs from worms intoxicated by Cry6Aa2 toxin were analyzed by quantitative real time PCR (qRT-PCR) (Fig. 2). Comparatively to control, 11 genes have shown significantly higher transcriptional levels (Fig. 2A–E), and well correlated with the 2-DE data, suggesting that the expressions of the 11 DEPs are mainly regulated at the transcriptional level. The left genes *pud-2*.1 and *pud-2.2* are gene duplications with exactly the same DNA sequence, encoding the same protein PUD-2; and so are *pud-1.1* and *pud-1.2*, both encode PUD-1 in worms^27^. PUD-1 and PUD-2 belong to PUD family which has not been characterized for its function^28^. Therefore, the transcriptional levels of both *pud-2* and *pud-1* in response to Cry6Aa2 toxin were tested together in this study. The qRT-PCR result indicated that at transcriptional level, both *pud-2* and *pud-1* were slightly down-regulated in the worms intoxicated by Cry6Aa2 (Fig. 2C). The transcriptional level of *pud-2* did not match the proteomic observation in which PUD-2.1 and PUD-2.2 were dramatically down-regulated (Table 1).

PUD-1 and PUD-2 are two proteins up-regulated in long-lived *daf-2* (loss-of function) mutant of *C*. *elegans*, which are abundantly expressed in the intestine and hypodermis, and form a heterodimer^28^. In addition, previous studies have shown that PUD-1 and PUD-2 are probably two downstream targets of DAF-16, a transcriptional factor in ILR signaling pathway^27,28^. All these findings suggest that PUD-1 and PUD-2 are associated with ILR signaling pathway. Therefore, the mRNA level changes of DAF-2 and DAF-16, the two main elements in the ILR signaling pathway, were also measured after N2 worms exposed to Cry6Aa2 toxin. The result showed that the mRNA level of *daf-2* was significantly increased by more than 13- fold, whereas the mRNA level of *daf-16* was drastically decreased by more than 27-fold (Fig. 2C). This result is line with the previous study in which DAF-2 negatively regulates DAF-16^19^, and suggests that ILR pathway is probably involved in the stress response of worms to Cry6Aa2 toxin. Curiously, the alterations of protein abundance of DAF-2 and DAF-16 were not be observed by proteomic analysis. This discrepancy of results between proteomic analysis and qRT-PCR is likely due to the limitations of sensibility and resolution of 2-DE and MS, or the experiment error.

The alterations of the expression level of DEPs at both mRNA level and protein level suggest that there are multiple biological processes involved in expression change of worms proteins caused by Cry6Aa2 toxin. To understand if there is a relationship between these DEPs and worms stress response to Cry6Aa2 toxin, the roles of these DEPs in worms stress response were subsequently investigated.

### Cry6Aa2 toxin promotes energy production of worms

**Figure 3:**
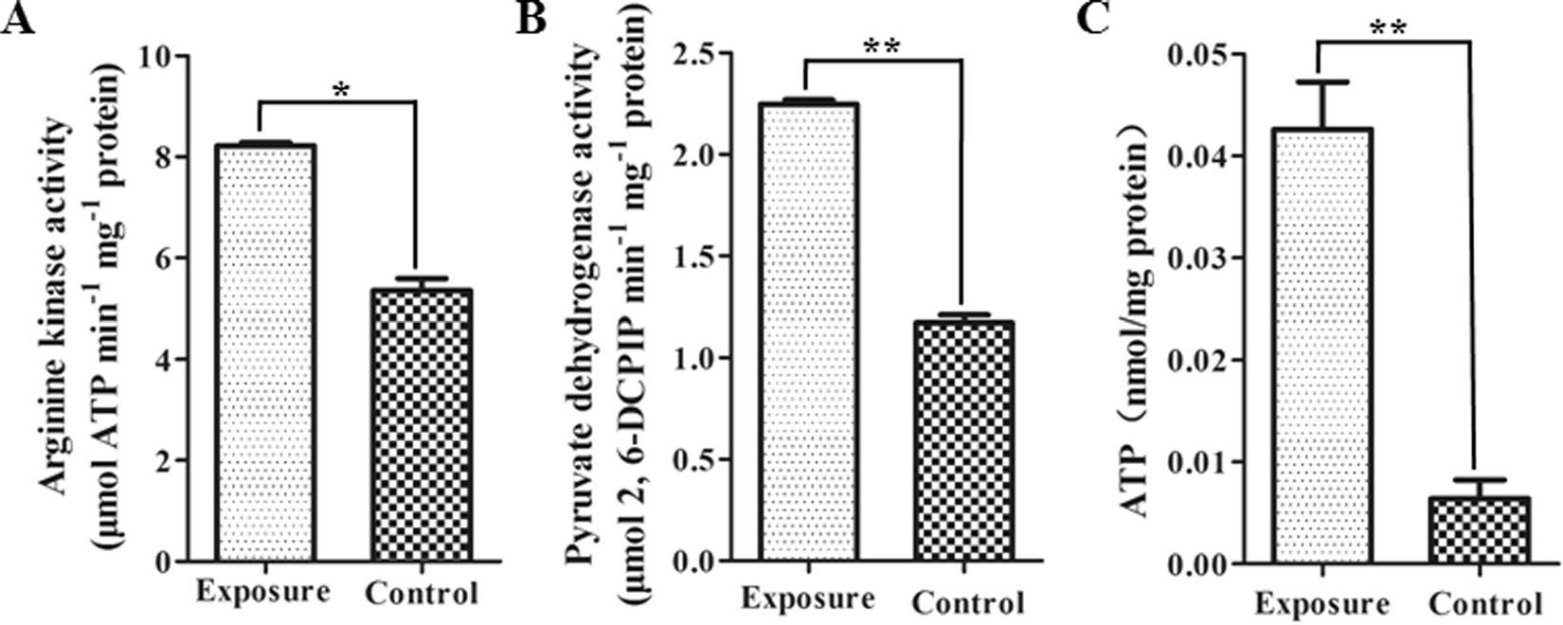
Effects of Cry6Aa2 toxin on the energy metabolism in *C*. *elegans*. (**A**) Effect of Cry6Aa2 toxin on the activity of arginine kinase; (**B**) effect of Cry6Aa2 toxin on the activity of pyruvate dehydrogenase E1; (**C**) effect of Cry6Aa2 toxin on the total cellular ATP. Data are shown as the mean ± SD. A single asterisk indicates *P* < 0.05, two asterisks indicate *P* < 0.01.

Proteomic observation and mRNA level test both indicated that five proteins (Table 1, Fig. 2B) involved in energy metabolism were significantly up-regulated when worms intoxicated by Cry6Aa2 toxin. Among which arginine kinase isoform b (AK-b) and pyruvate dehydrogenase E1 subunit (PDHB-1) are two key enzymes for energy production in two different pathways. AK (EC 2.7.3.3) is a specific phosphotransferase in invertebrate species, and it functions in the reversible synthesis of ATP from arginine phosphate and ADP^29^. PDHB-1 (EC 1.2.4.1) is one of the subunits of pyruvate dehydrogenase multienzyme complex (PDC) linking glycolysis pathway (from glycose to pyruvate) and tricarboxylic acid cycle (TCA, from acetyle-CoA to CO_2_ and H_2_O) in glucose glycolytic metabolism pathway^30^. In order to verify the impact of Cry6A toxin on energy production in worms, we tested the enzymatic activities of AK-b and PDHB-1, and then compared the change of cellular energy in worms in response to Cry6Aa2 toxin. In comparison with control, the enzymatic activities of both AK-b and PDHB-1 in response to Cry6Aa2 were increased by 1.53-fold (*P* < 0.01) (Fig. 3A) and 1.92-fold (*P* < 0.01) (Fig. 3B), respectively. This result indicates that Cry6Aa2 intoxication increased enzymatic activities of AK-b and PDHB-1 in worms, which agrees well with the transcription and expression levels of the two genes.

Cry6Aa2 toxin leads to the significantly increase of the enzymatic activities of AK-b and PDHB-1, related to metabolic energy production. Theoretically, the increased expressions of the two key enzymes responsible for energy production should significantly elevate ATP yield in *C*. *elegans*. Thereby, the total intracellular ATP level of *C*. *elegans* upon intoxication by Cry6Aa2 toxin was measured. As expected, the intracellular ATP level was dramatically increased 6.67-fold (*P* < 0.01) due to Cry6Aa2 toxin exposure (Fig. 3C). These findings suggest that exposure to Cry6Aa2 toxin can significantly enhance the energy production in worms. The boosted ATP production probably meets the increasing energy demand in different stress responses and innate immunity^31^.

### ILR signaling pathway contributes to the stress responses of worms towards Cry6Aa2 toxin

**Figure 4:**
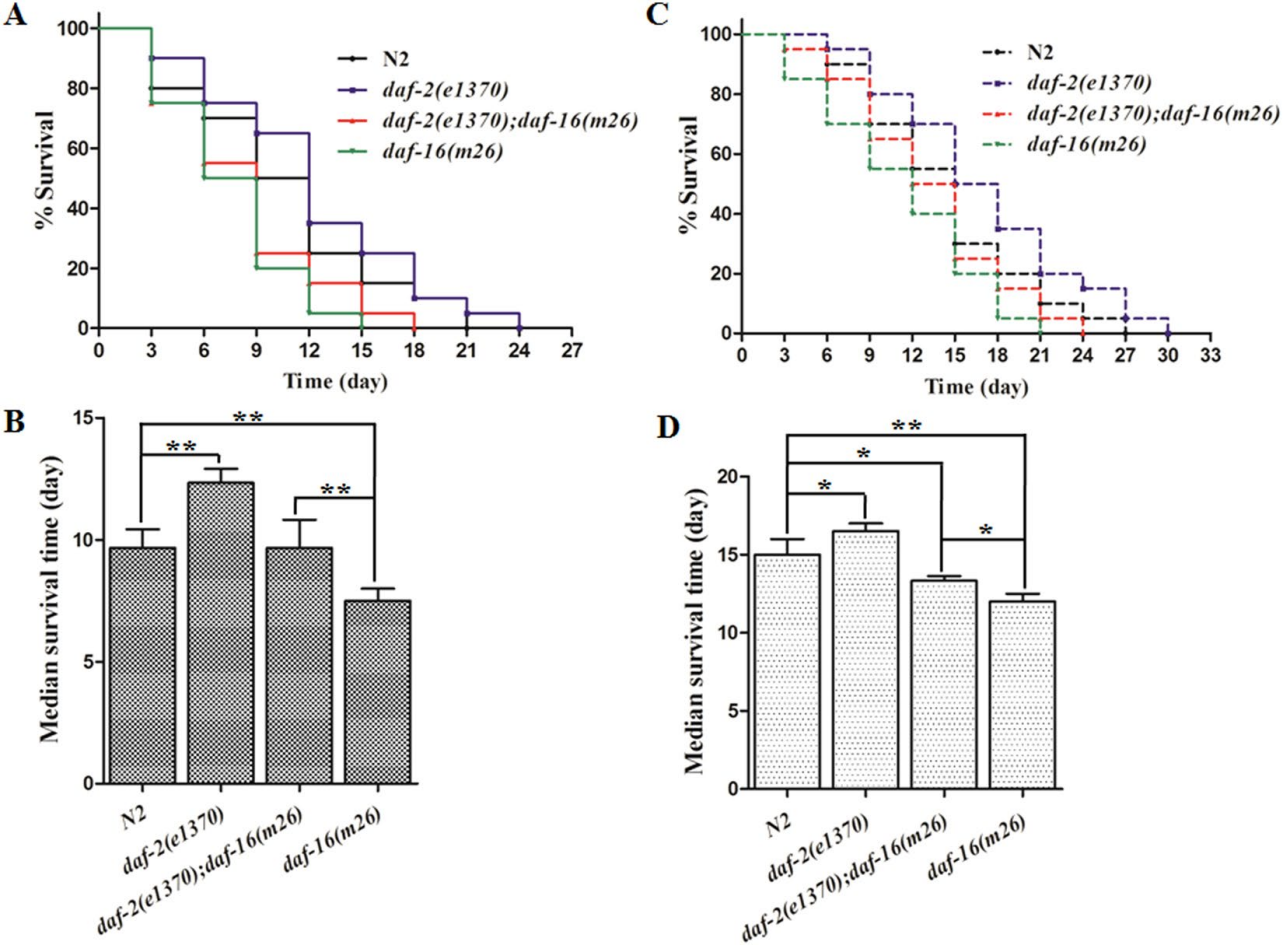
Resistance of *C*. *elegans* mutants related to ILR signaling pathway to Cry6Aa2 toxin. (**A**) The representative Kaplan-Meier Survival curves of *C*. *elegans* mutants exposed to Cry6Aa2 toxin. (**B**) Comparison of median survival times of *C*. *elegans* mutants upon exposure to Cry6Aa2 toxin. (**C**) The representative Kaplan-Meier Survival curves of *C*. *elegans* mutants unexposed to toxin. (**D**) Comparison of median survival times of *C*. *elegans* mutants without exposure to Cry6Aa2 toxin. The data were plotted using the Kaplan-Meier Survival curves and statistical significance was determined by Log-rank (Mantel-Cox) tests. Each point represents the mean and SD from three replicates. A single asterisk indicates *P* < 0.05, two asterisks indicate *P* < 0.01.

Exposure to Cry6Aa2 toxin caused significant proteins abundance changes of which are involved in ILR signaling pathway, such as Pud-1, Pud-2, Daf-2, and Daf-16 (Table 1, Fig. 2C). It suggests that ILR signaling pathway is probably involved in the defense response of *C*. *elegans* towards Cry6Aa2 toxin. Hence, we tested the median survival times of *C*. *elegans* mutants *daf-2*(*e1370*), *daf-16*(*m26*), two crucial genes in ILR signaling pathway, and double mutant *daf-2*(*e1370*);*daf-16*(*m26*), under exposure to Cry6Aa2 toxin. Upon exposure to Cry6Aa2 toxin (Fig. 4A,B) or not (Fig. 4C,D), *daf-2*(*e1370*) exhibited greater longevity compared to N2 worms and the other two mutants, *daf-16*(*m26*) and double mutant *daf-2*(*e1370*);*daf-16* (*m26*). In contrast, when not exposed to Cry6Aa2 toxin (increased by 10.0% compared to N2, *P* < 0.05) (Fig. 4D), the fraction extension in life-span of *daf-2*(*e1370*) exhibited greater degree under exposure to Cry6Aa2 toxin (increased by 26.8% compared to N2, *P* < 0.01) (Fig. 4B). This result suggests that *daf-2*(*e1370*) exhibited obvious resistance to Cry6Aa2 toxin compared to N2 worms, which agrees well with previous study, in which long-lived *C*. *elegans daf-2* mutants are resistant to bacterial pathogens including *Enterococcus faecalis*, *Staphylococcus aureus*, and *Pseudomonas aeruginosa* ^32^. However, the life-span of another single mutant *daf-16*(*m26*) exposed to Cry6Aa2 toxin was obviously shorter compared to N2 worms (*P* < 0.01) and even to double mutant *daf-2*(*e1370*);*daf-16*(*m26*) (*P* < 0.01) (Fig. 4B). Moreover, when unexposed to toxin, the life-span of *daf-16*(*m26*) is also shorter than that of N2 worms (*P* < 0.01) and the double mutant (*P* < 0.05) (Fig. 4D). This result suggests that *daf-16*(*m26*) is more hypersensitive compared to N2 worms and other mutants. Our result also supported the previous conclusion that DAF-16 directly responds to pathogens and thus contributes to immune defense^33^. Furthermore, the median survival time of double mutant *daf-2*(*e1370*);*daf-16*(*m26*) (9.7 ± 1.1 days) was modest compared to that of *daf-2*(*e1370*) (12.3 ± 0.6 days) and *daf-16*(*m26*) (7.5 ± 0.5 days) when exposed to Cry6Aa2 toxin (Fig. 4A,B), suggesting slighter degree of sensitivity to Cry6Aa2 toxin than *daf-16*(*m26*). This is most likely a consequence of *daf-2*(*e1370*) also suppressed the pathogen-sensitive phenotype of *daf-16* (*m26*)^32^.

### DIM-1 contributes to impairing locomotion defect of worms caused by Cry6Aa2 toxin

**Figure 5:**
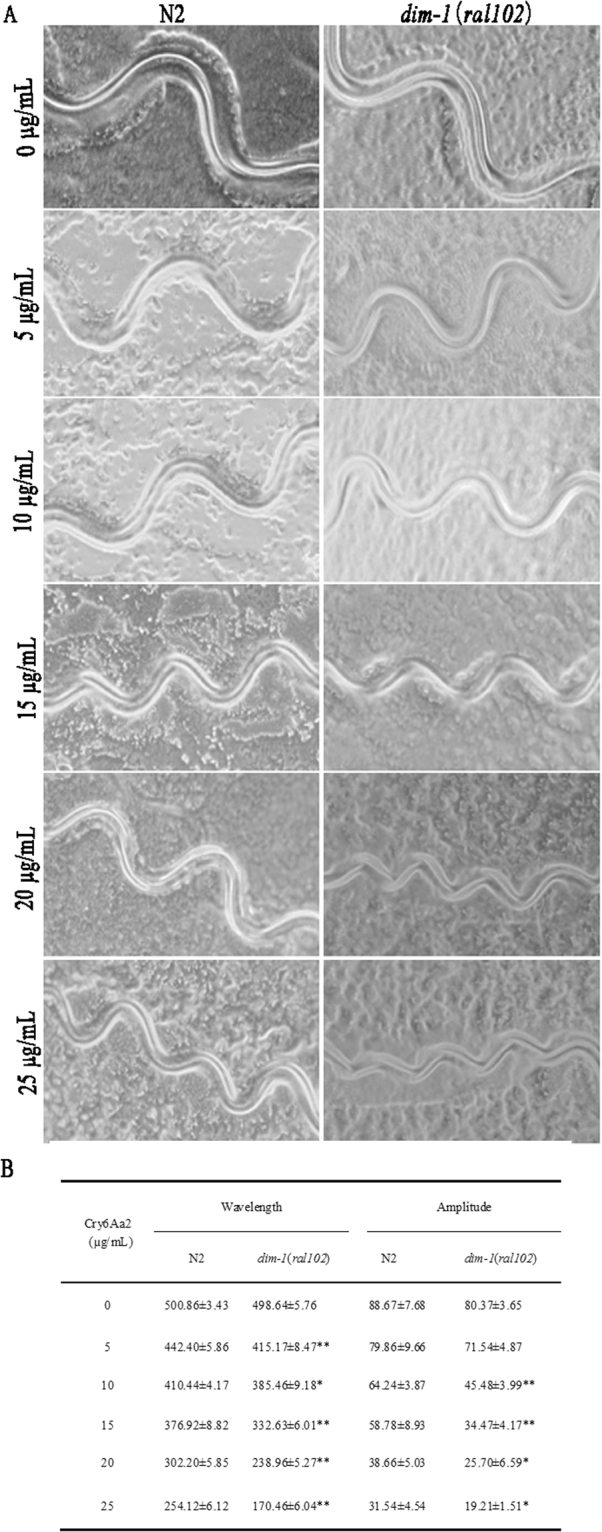
Locomotion abnormalities of wild-type N2 worms and *dim-1*(*ral102*) exposed to Cry6Aa2 toxin. (**A**) Track patterns inscribed by worms intoxicated by gradient of doses of Cry6Aa2 toxin. Tracks were carved into a bacterial lawn by worms. (**B**) Quantification of track abnormalities in serial doses of Cry6Aa2 toxin. Numbers cited are the average of 50 measurements per trial for 10 trials of worms. Scores are reported as mean ± SD. Asterisk indicates the statistical significance of track patterns inscribed by *dim-1*(*ral102*) intoxicated by Cry6Aa2 toxin relative to which of N2 worms intoxicated by the same concentration of toxin. A single asterisk indicates *P* < 0.05, two asterisks indicate *P* < 0.01.

Exposure to Cry6Aa2 toxin leads to obvious abundance increase of DIM-1 isoform b (Table 1, Fig. 2D), a disorganized muscle protein 1^34^. In order to investigate the biofunction of *dim-1* gene in the defense response of worms, the movement tracks of mutant *dim*-*1*(*ra102*) and N2 worms intoxicated by Cry6Aa2 toxin were recorded in the background of *E. coli* lawn. Moreover, the impacts of Cry6Aa2 toxin on mobility were further analyzed by measuring the amplitude and wavelength of the inscribed sinusoidal wave path. When not exposed to Cry6Aa2 toxin (0 μg/mL), there was no obvious difference observed between the track patterns generated by *dim-1*(*ra102*) and N2 worms. Whereas, the track patterns generated by *dim-1*(*ra102*) and N2 under exposure to Cry6Aa2 toxin exhibited apparently difference (Fig. 5A). In general, the wave paths of both N2 and *dim-1*(*ral102*) exhibited dose-dependent reduction when exposed to Cry6Aa2 toxin (Fig. 5A). The influence of Cry6Aa2 toxin on locomotion curve of *dim-1*(*ra102*) exhibited greater extent than on N2, for both the wavelength and amplitude (Fig. 5B). These data suggest that *dim-1*(*ra102*) motility is more hypersensitive to Cry6Aa2 toxin than that of N2 worms, implying the crucial role of DIM-1 in mitigating locomotion defect of worms.

### LEC-6 is involved in the stress response of worms towards Cry6Aa2 toxin, whereas LEC-2 is not

**Figure 6:**
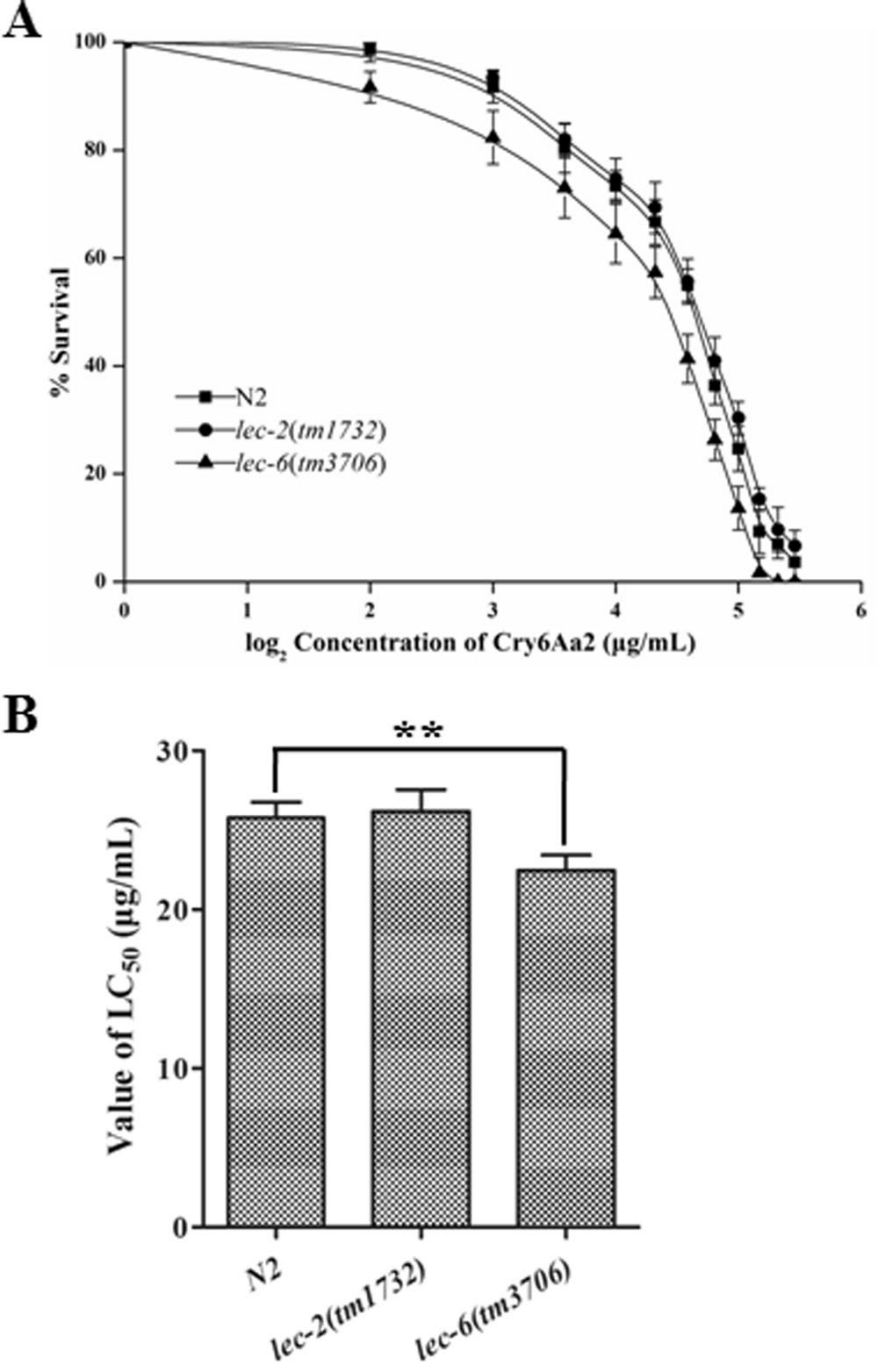
Hypersensitivity comparison of *C*. *elegans* mutants related to galectin to Cry6Aa2 toxin. (**A**) Dose-dependent mortality assays of Cry6Aa2 against mutants. The percentages of alive worms as a function of Cry6Aa2 concentration were plotted using nonlinear regression. Each point represents the mean and standard deviation from three independent experiments with five replicates. (**B**) Comparison of the LC_50_ values of Cry6Aa2 against N2 worms and mutants. Data were showed as mean ± SD. Two asterisks indicate *P* < 0.01.

Two galectins, LEC-2 and LEC-6, were differentially expressed upon exposure to Cry6Aa2 toxin (Table 1, Fig. 2E). In order to further verify the function of galectin in the response of worms against Cry6Aa2 toxin, the susceptivities to Cry6Aa2 toxin of the two corresponding gene mutants, *lec-2*(*tm1732*) and *lec-6*(*tm3706*), were compared with that of N2 (Fig. 6A). The result showed that *lec-6*(*tm3706*) is more sensitive to Cry6Aa2 (*P* < 0.01), whereas, *lec-2*(*tm1732*) exhibited nearly equivalent susceptivity with N2 (Fig. 6B). This result suggests that *lec-6* contributes to the defense response of *C*. *elegans* against Cry6Aa2 toxin, and no defense response is contributed by *lec-2*.

## Discussion

This study focused on the proteomic alternations induced by Cry6A toxin in *C*. *elegans*. Our study showed that Cry6A toxin is significantly involved in altering the protein profile of *C*. *elegans*. A total of 12 DEPs were detected and characterized by 2-DE followed MALDI-TOF/TOF MS. Among the 12 DEPs, the abundances of 11 DEPs were significantly up-regulated, and only one DEP (PUD-2) was down-regulated. It is notable that the HSP-6 and PUD-2 in the 2-DE gel were represented by more than one spot (Fig. S1, Table 1). The same protein is represented by more than one spot is likely attributed to different isoforms, post-translational modifications (PTMs), or alternative mRNA splice forms of this protein^35^. Furthermore, the abundance changes of the 12 DEPs and other relevant proteins (PUD-1, DAF-2, and DAF-16) on transcriptional level were tested by qRT-PCR. The results indicated that the changes of most DEPs on transcriptional level correlated well with those on expression level. However, the mRNA level of *pud-2* did not match the proteomic observation (Table 1, Fig. 2C). Indeed, the transcription level of a gene does not be necessarily correlated with the level of corresponding protein, because the abundance of a protein depends not only on transcription rate of the gene expression alone^36,37^.

Based on the *C*. *elegans* genome database and previous reports, the identified proteins were annotated for their specific biological functions, including stress response, energy production metabolism, ILR signaling pathway, and muscle assembly. The significant alterations on the expression abundances suggest that these proteins play crucial roles in the interaction of *C*. *elegans* and Cry6A toxin.

Living cells upon confronting adverse environmental stimulator, physiological stressor and even bacterial infection, can exert a rapid molecular response to promote cell adaptation for survival. HSP family represents one such example^38^. HSPs are highly evolutionarily conserved molecular chaperones, which are ubiquitously found in subcellular compartments, cells, tissues, and give immediate responses during any stress, tissue damage, or bacterial infection^39,40^. HSP exerts physiological effect by modulating and assisting folding of newly synthetic polypeptides as well as maintenance of the proper conformation of protein, preventing protein denaturation or aggregation^41,42^. Furthermore, the role of HSP is very crucial in pathological conditions by rectifying structurally denatured proteins, and degradation of proteins in failure conditions of refoldings^43,44^. This idea is in consistent, that Cry6A intoxication might alter the expression level of HSP, as three HSPs (HSP-6, HSP-25, and HSP-12.2) in wild-type N2 worms were remarkably up-regulated in response to Cry6Aa2 toxin (Fig. 1, Table 1). This result suggests that HSPs play key role in the stress response of *C*. *elegance* against Cry6Aa toxin. Many previous reports are in agreement with our result about the roles of HSP in bacterial infection and immunity^40^. In view of the universality of HSP’s function in stress responses, the contributions of the identified three HSPs in the defense response of *C*. *elegans* against Cry6A toxin were not further confirmed in this study.

Cry6Aa2 toxin increased abundances of five proteins related to energy metabolism (ENOL-1 isoform a, ENOL-1 isoform b, AK-b, PDHB-1, and cytb-c1 complex) (Table 1), which means the intoxication by Cry6A toxin altered worms energy metabolism. Moreover, the transcriptional level analyses of the five genes in response to Cry6A toxin revealed the significant increases which correspond to those on proteomic level (Fig. 2B). All these results strongly suggest that the intoxication by Cry6A toxin increases the energy metabolism in worms. The increased expressions of these key enzymes responsible for energy production suggest an increase of ATP yield in *C*. *elegans* under exposure to Cry6Aa2 toxin. The subsequential measurement of total intracellular ATP level in *C*. *elegans* upon intoxication by Cry6Aa2 toxin indicated that Cry6A toxin dramatically elevated the ATP yield in *C*. *elegans* (Fig. 3C).

Of the five proteins related to energy production, AK-b and PDHB-1 are two crucial enzymes involved in two main pathways for energy production in worms. AK is a specific phosphotransferase in invertebrates^45^. It catalyzes the reversible phosphorylation of arginine by Mg-ATP to form phosphoarginine^45^. When there is a burst cellular activity in invertebrates, phosphoarginine can function as ATP buffer, permitting maintenance of high ATP level^46^. Many previous studies have demonstrated that AK in invertebrate plays important role in defense response to oxidative stress^47^, hypoxic stress^48^, infection of virus^49^, heavy-metal stimulation^50^, and so on. Our study revealed that, the expression of AK-b was dramatically induced by exposure to Cry6Aa2 toxin, which shows consistency of the idea that increased expression of arginine kinase contributes to the defense response of *C*. *elegans* towards Cry6A toxin.

PDHB-1 is one of the subunits of PDC that is present in most prokaryotic and eukaryotic organisms, and plays crucial role for energy production^30^. The role of PDC in glycolytic metabolism is central by linking glycolysis pathway and TCA via converting pyruvate to acetyl-CoA^30^. PDC comprises of three catalytic subunits: (i) pyruvate dehydrogenase (E1) catalyzing the decarboxylation of pyruvate followed by reductive acetylation of lipoyl moieties covalently linked to the dihydrolipoamide acetyltransferase (E2); (ii) E2 catalyzing the formation of acetyl-CoA; (iii) dihydrolipoamide dehydrogenase (E3) reoxidizing the reduced lipoyl moieties of E2 with the consequent reduction of NAD^+^ to NADH^51^. PDC is the only known pathway in most eukaryotes to generate acetyl-CoA from pyruvate, and is tightly regulated by reversible phosphorylation/dephosphorylation that requires additional regulatory enzymes, pyruvate dehydrogenase kinase (PDK) and phosphopyruvate dehydrogenase phosphatase (PDP)^30^. This regulation plays critical role in energy metabolism to meet the specific metabolic and energetic demands of different tissues, under different physiological conditions. This study concludes the increase in expression of PDC upon exposure to Cry6Aa2 toxin is the contribution to immunity of worm.

The above mentioned results suggest that two major energy production pathways in worm, such as AK pathway and glycolytic metabolism pathway, are activated by exposure to Cry6Aa2 toxin and, results in boosted ATP production. Previously it is reported by the studies that increase in metabolic energy is a typical adaptive response under hypoxia-induced stress^52^, heavy metal-induced neurotoxicity^53,54^, and extremely low-frequency electromagnetic field exposure^55^. It is a ubiquitous mechanism existed in animals and plants^31^. Consistent with this idea, we propose that energy enhancement might be conducive to Cry6A toxin-induced stress response and innate immunity in worms. Although previous studies have investigated the global cellular responses of *C*. *elegans* to pathogenic Bt^56^ and pore-forming toxin Cry5B^26^, this study is the first discovery which describes that the metabolic energy is clearly involved in the stress response of *C*. *elegans* towards nematicidal crystal protein. This finding provides new evidence in support of the critical function of energy in host stress responses, and will also contribute to in depth understanding the highly complex host-pathogen interactions.

DAF-2 and DAF-16 are two main elements in ILR signaling pathway. DAF-2 is a transmembrane tyrosin kinase, and DAF-16 is a forkhead/winged helix-related transcription factor of the FOXO family^57,58^. DAF-2 negatively regulates DAF-16 through phosphorylation of the phosphatidylinositol-3-OH kinase (PI3K) encoded by *age-1*, and subsequent activation of four serine/threonine kinases: PDK-1 (the PI3K-dependent kinase homolog), the Akt/PKB homologs AKT-1 and AKT-2, and SGK-1 (serum- and glucocorticoid-inducible kinase homolog), ultimately leading to phosphorylation and cytoplasmic retention of DAF-16^58–61^. Down-regulation or inactivation of DAF-2 could lead to the translocation of DAF-16 into the nucleus. Upon nuclear translocation, DAF-16 regulates a large number of genes involved in the regulation of antimicrobial, detoxifying, stress-responses, subsequently conferring increased longevity and stress resistance^62–64^. All these findings elucidate that the ILR signaling pathway contributes to nematode stress response and immunity^65^, and responses to environmental stimuli^66^. Moreover, many reports have shown that the mutants related to ILR signaling pathway exhibit increased worm survival in the presence of pathogens^65,67^. Our results support this by observing the significant changes at the transcription level of DAF-2 and DAF-16, and it has been proven to be closely related with hypersensitivity of the worms towards Cry6Aa2 toxin. This is a clear proof about ILR signaling pathway involvement in stress response of worms against Cry6Aa2 toxin. In addition, Cry6Aa2 toxin dramatically decreased the expressions of PUD-1 and PUD-2, which are probably the targets of DAF-16 in ILR signaling pathway^27^. Our result correlates with previous studies, in which deletion of *pud-1* and *pud-2* was associated with protections against amyloid β-peiptide^28^, and high concentration of CO_2_^68^. Moreover, remarkable decreases of PUD-1 and PUD-2 were also observed in many mutants in response to different stimuli^69–72^. Hence, we propose that Cry6Aa2 toxin down-regulates PUD-1 and PUD-2 expression *via* ILR signaling pathway, PUD-1 and PUD-2 are probably the downstream targets of transcription factor DAF-16 of ILR signaling pathway in response to Cry6Aa2 toxin. The correlations of PUD-1 and PUD-2 with the stress response of worms towards Cry6Aa2 toxin should be confirmed in future study.

DIM-1, a novel immunoglobulin superfamily protein containing three immunoglobulin-like repeats or domains in *C*. *elegans*, is necessary for maintaining bodywall muscle integrity^34^. DIM-1 localizes to the region of the muscle cell membrane around and between the dense bodies^73^. After exposure of N2 worms to Cry6Aa2 toxin, DIM-1 (isoform b) was significantly up-regulated (Table 1, Fig. 2D). Previously it was studied in mutant hermaphrodite *unc*-12 that loss or reduction of *dim*-*1* gene function can suppress the severe muscle disruption and paralysis^34^, also can mitigate locomotion defects in mutants related to genes encoding integrin muscle attachment complex components^74^. Our previous study showed that wild-type worm N2 locomotion abnormality is due to Cry6A toxin intoxication^75^. In this study, we found that Cry6Aa2 toxin leads to obvious up-regulation of DIM-1, implying that DIM-1 is presumably involved in the mitigation of locomotion defects caused by pathogens. Thereby, we investigated the impact of Cry6Aa2 toxin on locomotion of *dim-* *1* mutant. The result showed that Cry6Aa2 toxin caused greater movement impairments in *dim-1* mutant compared to wild-type N2. This result suggests that DIM-1 can mitigate the locomotion defects caused by toxin. Unfortunately, there is still no experimental evidence supporting motility regulation of *dim-1*. Further investigation should be needed to understand the function of *dim-1* in motility regulation.

Galectins are a group of lectins that bind various β-galactoside-containing carbohydrate chains attached to proteins and lipids^76^. By interacting with glycoconjuagtes that contain β-galactoside units, such as galactose-β1,4-N-acetylglucosamine (Galβ1-4GlcNAc), galectins play important roles in various biological events such as development, immunity and cancer defense^77,78^. More than 10 galectin genes have been characterized from the genome of *C*. *elegans* ^79^, some of which have been confirmed to confer diverse range of stress responses in worms. For example, LEC-8 contributes to worm defense against Cry5B toxin intoxication by competitive binding to target glycolipid molecules^80^; LEC-10^81^ and LEC-1^82^ confers worm some protection against oxidative stress. In the current study, two galectins, LEC-2 and LEC-6, were up-regulated both on mRNA and protein levels upon exposure to Cry6Aa2 toxin (Table 1, Fig. 2E), implying galectin is involved in the defense response of worm against Cry6A toxin. However, assays of hypersensitivity to Cry6Aa2 showed that loss-of-function mutant *lec-2*(*tm1732*) exhibited comparable sensitivity with N2, and *lec-6*(*tm3706*) exhibited more hypersensitive than N2. This result suggests that only LEC-6, not LEC-2 plays role in the defense response of worms towards Cry6Aa2 toxin (Fig. 6). Due to the discrepancies on structure and glycan ligand, different galectin in *C*. *elegans* might play different role^77^. The detailed roles of LEC-2 and LEC-6 in defense response to Cry6A need future investigation.

Collectively, the current study revealed that different stress responses were involved in the stress responses of worms towards Cry6A toxin, including HSPs, ILR signal pathway, galectins, DIM, and energy metabolism. Of which, HSP, DIM-1, and energy production were for the first time observed to participate in the defense responses of worms against crystal proteins produced by Bt, although their detailed functions and mechanisms in stress responses remain obscure. Moreover, this study has also shed light on the downstream mechanisms involved in the *C*. *elegans* immune response to Cry6A toxin. It is worth noting that the stress response of worms against Cry6A does not exclusively rely on the observed pathway in this study, because of the limitations of sensibility and resolution of 2-DE and MS. In addition, due to the distinct architecture and action mechanism, extremely high toxicity to plant-parasitic nematode^83^, Cry6A toxin represents a promising candidate for nematicidal agent. Understanding the nematode defense responses could provide novel insights on delaying and overcoming the potential resistance of nematode in long-term and large-scale usage of Bt for nematode biocontrol.

## Materials and Methods

### Bacterial strains and culture conditions

Two recombinant *E*. *coli* strains YEC-10 and YEC-11 were constructed in previous study^75^. The YEC-10 is *E*. *coli* BL21 (DE3) harboring *cry6Aa2* (GenBank acc. no. AF499736) cloned in expression vector pGEX-6p-1, and YEC-11 is *E*. *coli* BL21 harboring empty vector pGEX-6p-1. YEC-10 was induced to express Cry6Aa2 toxin by 0.1 mM isopropyl β-D-1-thiogalactopyranoside (IPTG) at 30 °C in LB medium. The YEC-11 was induced expression in parallel and used as control. Recombinant Bt strain BMB171-15 is the acrystalliferous mutant strain BMB171 harboring *cry6Aa2* cloned in Bt-*E*. *coli* shuttle vector pHT304^75^, and was cultured at 30 °C in ICPM medium for preparation of Cry6Aa2 toxin^75^.

### *C*. *elegans* maintenance

Strains of *C*. *elegans* used in this study were wild-type Bristol strain N2, *daf-2*(*e1370*), *daf-16* (*m26*), double mutant *daf-2*(*e1370*); *daf-16* (*m26*), *dim*-*1*(*ra102*), *lec-2*(*tm1732*), and *lec-6*(*tm3706*). All *C*. *elegans* strains were obtained from the Caenorhabditis Genetics Center (University of Minnesota, Minneapolis, MN, USA). *C*. *elegans* strains were maintained at 20 °C on nematode growth medium (NGM) plates using *E. coli* OP50 as the food source^84^. L4 stage larval worms were obtained by growing synchronized L1 larval stage worms on NGM plates seeded with *E*. *coli* OP50 at 20 °C for 44 h^85^.

### Toxicity assay

For 2-DE analysis, Cry6Aa2 toxin was expressed by induction of IPTG in *E*. *coli* strain YEC-10^75^. As control, *E*. *coli* YEC-11 was induced in parallel. Toxicity assays for 2-DE based on visual comparison intoxication were performed at 20 °C on plates with *E*. *coli*-expressed Cry6Aa2 (YEC-10)^75^. After induction by IPTG, YEC-10 and YEC-11 were harvested by centrifugation and resuspended in S medium^84^ with OD6_00_ of 2.0, respectively. The suspensions of YEC-10 and YEC-11 were mixed at a ratio of 1:3 (v/v)^24^. 100 μL of the mixture was spread on NGM plate (diameter of 9 cm), and allowed to dry overnight at 20 °C. 200 L4 stage N2 worms were transferred to a plate, and incubated at 20 °C for 2 days. This dosage of Cry6Aa2 (25%) and intoxication time can mildly intoxicates wild-type N2, which facilitates identification of alive worms that are intoxicated by Cry6Aa2 toxin^75^. For control, L4 worms were intoxicated by YEC-11 strain in the same way.

For lethal concentration assays, Bt strain BMB171-15 was cultured to express Cry6Aa2 crystal protein toxin as previously described^75^. Cry6Aa2 toxin was prepared and purified according to the method described by Griffitts (2001)^14^, and dissolved in 20 mM 4-(2-hydroxyethyl)-1-piperazineethanesulfonic acid (HEPES) (pH8.0) prior to use. Protein concentration was determined by the Bradford method^86^. The hypersensitivities of N2 worms and mutants to Cry6Aa2 toxin were performed in a 48-well plate format as described previously^75^. Concentrations of Cry6Aa2 toxin were set up in quintuplicate for each assay, and each assay was carried out independently in triplicates. Approximately 1000 worms were scored for each strain in the calculation of the LC_50_ value.

### 2-DE and image analysis

After intoxication by Cry6Aa2 toxin, worms were harvested and washed thoroughly with M9 buffer^84^ to remove the surface bound bacteria, and homogenized immediately in liquid nitrogen to extract worms global protein for 2-DE. The detailed methods of *C*. *elegans* global protein preparation and 2-DE analysis were carried out as previously described^87^. Protein concentration was determined by the Bradford method^86^. Each sample containing 200 µg total protein in 350 µL of rehydration solution (8M urea, 2M thiourea, 4% (w/v) CHAPS, 0.5% (v/v) IPG buffer, 60 mM DTT, 40 mM Tris base, and trace amount of bromophenol blue) was isoelectrically separated on an 18-cm immobilized IPG strip (Bio-Rad, Pennsylvania, USA) with a linear pH gradient of 4–7. The second-dimensional separation was carried out on a 12% SDS-PAGE gel, followed by Coomassie Brilliant Blue G-250-staining visualization and image acquisition. Gel images were analyzed with ImageMaster 2D Platinum 6.0 (GE Healthcare, Uppsala, Sweden) according to the manufacture’s protocol. The protein spots with significant differences at fold change over 2.0 across three analytical gels were picked and subjected to tryptic digestion and mass spectrometric (MS) analysis.

### Tryptic in-gel digestion and MALDI-TOF/TOF MS analysis

The protein spots with significant difference were excised for tryptic in-gel digestions as previously described^88^. The digested peptides were analyzed on an ABI 4800 plus MALDI TOF/TOF MS (Applied Biosystems, Foster City, CA, USA). The MS and MS/MS data were interpreted using GPS Explorer software V3.6 (Applied Biosystems), and submitted to MASCOT search engine V2.1 (Matrix Science, London, UK) for protein identification^89^. Protein function was cited from the WormBase database (http://wormbase.org), the ExPASy server (http://kr.expasy.org) and the National Center for Biotechnology Information (http://www.ncbi.nlm.nih.gov) database.

### RNA isolation and quantitative real time PCR (qRT-PCR) analysis

L4 stage *C*. *elegans* was washed repeatedly with M9 buffer to remove adhered bacterial, and were flash frozen. Total RNA was extracted from worms with Trizol reagent (Invitrogen, Carlsbad, CA, USA) according to the manufacturer’s instructions. DNase I (New England Biolabs, Ipswich, MA, USA) was used to remove the contaminant of genomic DNA, followed by column purification using RNeasy Mini Kit (QIAGEN, Valencia, CA, USA). The RNA was solubilized in RNase-free double distilled water (0.5-1.0 μg/μL).

qRT-PCR was performed on a ABI 7500 Real-Time PCR Detection System (Applied Biosystems) using Power SYBR^®^ Green RNA-to-C_T_^TM^ 1-Step Kit (Applied Biosystems). Cycling conditions were 48 °C for 30 min (reverse transcription) and 95 °C for 10 min (activation of AmpliTaq, Gold^®^ DNA Polymerase), followed by 40 cycles of 95 °C for 15 s (denature), 60 °C for 1 min (anneal/extend). The expression levels were normalized to *act-1* ^90^ for DEPs coding genes, and the relative expression was calculated as 2^−ΔΔCt^ (cycle threshold). Nuclease-free double distilled water was used as the template in the control. The specific primer pairs (forward and reverse) for the DEPs coding genes and primer pair for reference gene (*act-1*) were listed in Table S1.

### Activity assay of arginine kinase (AK-b)

Worms were harvested and washed with 50 mM Tris-acetate buffer (pH8.0), and were homogenized immediately by grinding in liquid nitrogen. The homogenates were dissolved in buffer of 50 mM Tris-acetate (pH8.0), and were centrifuged (12,000 rpm) for 30 min at 4 °C to obtain a crude enzyme solution. Coomassie Brilliant Blue assay was used to determine the amount of protein^86^. By using an established ternary heteropolyacid system, the catalytic activity of AK-b in the forward direction (formation of phosphoarginine) was determined^91^. The reaction was performed in 1-mL volume containing 2 mM L-arginine, 4 mM ATP, 5 mM Mg-acetate, 50 mM Tris-acetate (pH8.0)^91^. Reaction was allowed to continue for 10 min at 30 °C. The enzymatic activity of AK-b was expressed as the consumption rate of ATP (µmol min^−1^ mg^−1^ crude protein). ATP was measured using a firely luciferase-based ATP assay kit (Beyotime, Jiangsu, China) according to the manufacturer instructions. Luminance (relative to luminescence units, RLU) was measured by a GloMax TM 20/20 Luminometer (Promega, Madison, WI, USA).

### Activity assay of pyruvate dehydrogenase E1 subunit (PDHB-1)

To assay the activity of PDHB-1, the crude enzyme was extracted from worms, and protein amount was determined using the same methods described in the method of AK-b activity assay. The PDHB-1 activity was measured in the model reaction with 2, 6-dichlorophenolindophenol (2, 6-DCPIP) by monitoring the reduction of 2, 6-DCPIP at 600 nm^92^. The reaction was performed in a 1-mL test volume containing 50 mM KH_2_PO_4_ (pH7.0), 1mM MgCl_2_, 2.0 mM sodium pyruvate as substrate, 0.2 mM ThDP (thiamin diphosphate), and 0.1 mM 2,6-DCPIP. Reaction was allowed to continue for 15 min at 30 °C. The enzymatic activity of PDHB-1 was expressed as the reduction rate of 2, 6-DCPIP (µmol min^−1^ mg^−1^ crude protein).

### ATP assay

Total intracellular ATP of worms was measured using the same method described in the activity assay of arginine kinase. Briefly, nematode lysate was centrifuged at 12000 g for 10 min at 4 °C after lysis. In a microtube, 200 µL of each supernatant was mixed with 100 µL ATP detection working dilution. Subsequently, RLU was assayed.

### Worm motility assay

Worm motility assay was carried out as described previously^75^. Briefly, worms were intoxicated by gradient concentrations of Cry6Aa2 toxin for 4 h at 20 °C. For control, worms were treated with no toxin under the same conditions. Thereafter, individual L4 worms were transferred to fresh NGM plates with *E*. *coli* OP50 lawn and allowed to cut tracks for 10–20 min before paths were photographed for measurement. The locomotion defect was quantitated by measuring the amplitude (the height between peak and valley) and wavelength (the distance between successive peaks) in the path. For each treatment, 10 trials were conducted and 50 measurements were recorded per trial.

### Lifespan assay

Worm lifespan assays were performed at 20 °C as described previously^93^ with some modifications. Briefly, worm populations were synchronized as mentioned above. For each lifespan assay, worms were transferred every other day to new NGM plate seeded with *E*. *coli* YEC-10 and YEC-11 to eliminate confounding progeny, and were marked as dead or alive. Worms were scored as dead if they did not respond to repeated prodding with a platinum pick. Worms were censored if they crawled off the plate or died. For each lifespan assay, 90 worms were used in 3 plates (30 worms per plate). The data were plotted with the Kaplan-Meier Survival curves, and statistical significance was determined by Log-rank (Mantel-Cox) test. The hypersensitivities of each worm strain to Cry6Aa2 toxin were assessed by calculating the median survival times (required to kill 50% of the *C*. *elegans* population). Lifespan assays were repeated at least three times and showed similar trends in relative lifespan effects.

### Data analysis

All experiments were repeated a minimum of three times. LC_50_ values were calculated by PROBIT analysis. Toxicity assays are presented graphically using nonlinear regression performed with GraphPad Prism 6.0 (GraphPad Prism Software Inc., San Diego, CA, USA). Statistical analysis between two values was compared with an unpaired two-tailed Student’s t-test. For multiple-group comparisons, ANOVA was performed followed by Tukey post hoc test. Lifespan data was analyzed with Kaplan-Meier Survival curves followed by Log-rank (Mantel-Cox) test. Descriptive data are presented as the mean ± standard deviation (SD). Differences were considered significant at *P* < 0.05.

### Data availability

All data generated or analyzed during this study are included in this published article (and Supplementary Information Files).

## Electronic supplementary material

Supplementary information
