# Supplementary material for: A Proteomic Analysis Provides Novel Insights into the Stress Responses of Caenorhabditis elegans towards Nematicidal Cry6A Toxin from Bacillus thuringiensis
Source: Sci Rep. 2017 Oct 26;7:14170. doi: 10.1038/s41598-017-14428-3 (PMC5658354; doi:10.1038/s41598-017-14428-3)
Supplement: Supplementary file 1 — Supplementary information [file 41598_2017_14428_MOESM1_ESM.doc]

Supplementary information

**Title: A Proteomic Analysis Provides Novel Insights into the Stress Responses of *Caenorhabditis elegans* towards Nematicidal Cry6A Toxin from *Bacillus thuringiensis***

**Bing Wang^*1^, Haiwen Wang^*1^, Jing Xiong^1^, Qiaoni Zhou^1^, Huan Wu^1^, Liqiu Xia^1^ Lin Li^2^ & Ziquan Yu^1†^**

**^1^**State Key Laboratory of Developmental Biology of Freshwater Fish, College of Life Science, Hunan Normal University, Changsha 410081, P. R. China

**^2^**State Key Laboratory of Agricultural Microbiology, College of Life Science and Technology, Huazhong Agricultural University, Wuhan 430070, P. R. China

**^*^**These authors contributed equally to this work

**Correspondence to:**

**^†^**Ziquan Yu, College of Life Science, Hunan Normal University, 36 Lushan Road, Changsha 410081, P. R. China

E-mail: [zq01yu@gmail.com](mailto:zq01yu@gmail.com)

Table S1 Primer pairs for qRT-PCR amplification in this study

^a^F, forward primer; R, reverse primer

| Primer^a^ | Sequence  (5’ 3’) | Fragment size (bp) |
| --- | --- | --- |
| ACT-1-F | CGGAGGAACCACCATGTACC | 122 |
| ACT-1-R | ATCCAGACGGAGTACTTGCG |
| DIM-1-F | GATTCTGTCAGAGTTGGCAATG | 166 |
| DIM-1-R | GACGGACAAGTTATGGTTATGG |
| DAF-2-F | TGCTGCCGAGTACGCTGTCA | 153 |
| DAF-2-R | GCAAGTGGTGTTCGACCAAC |
| DAF-16-F | GAGAGCATTGATGGGCTCCC | 188 |
| DAF-16-R | TGGAGAAACACGAGACGACG |
| PUD-1-F | AAATCTTCAAAAGTGTCGCC | 156 |
| PUD-1-R | CTGATGAATTTCGCATGCTC |
| PUD-2-F | GTGAAATTGGAGCCAAACCA | 168 |
| PUD-2-R | CACCGAGAATACGAACCTTTCC |
| HSP-6-F | TCATCAACGAGCCAACTGCT | 157 |
| HSP-6-R | ATCTCCGTTGGTGGACTTGA |
| HSP-12.2-F | AATCGAGGTCAAGGTTTCCG | 146 |
| HSP-12.2-R | AGGTGGGATTTGACGGTGGA |
| HSP-25-F | CACGCCAAACACGAGGAGAA | 135 |
| HSP-25-R | CGACAGTGAGCACGCCATCA |
| PDHB-1-F | AAGGGATTGTGGAAGAAGCA | 153 |
| PDHB-1-R | GATCGATGGCTTGCATGGAA |
| UCR-1-F | TCGAGCGATTGATCCACAAG | 176 |
| UCR-1-R | GGAGAACATCGGCGAGGATA |
| ENOL-1-a-F | CACGGTGCCACTTCCATTCA | 152 |
| ENOL-1-a-R | CACGGTGCCACTTCCATTCA |
| ENOL-1-b-F | CCACGGTGCCACTTCCAT | 150 |
| ENOL-1-b-R | TGGCTGCTTCGATTGACTCTGT |
| AK-b-F | ATTCAACCCATGCCTT | 136 |
| AK-b-R | ATCTTGGATTTCCTTG |
| LEC-6-F | TGCAAGGATTCTCCAA | 165 |
| LEC-6-R | AACCTCCTCCGCTGGT |
| LEC-2-F | ACTCTCCTCCATCACA | 147 |
| LEC-2-R | ATTCCAAAGACAGTAA |


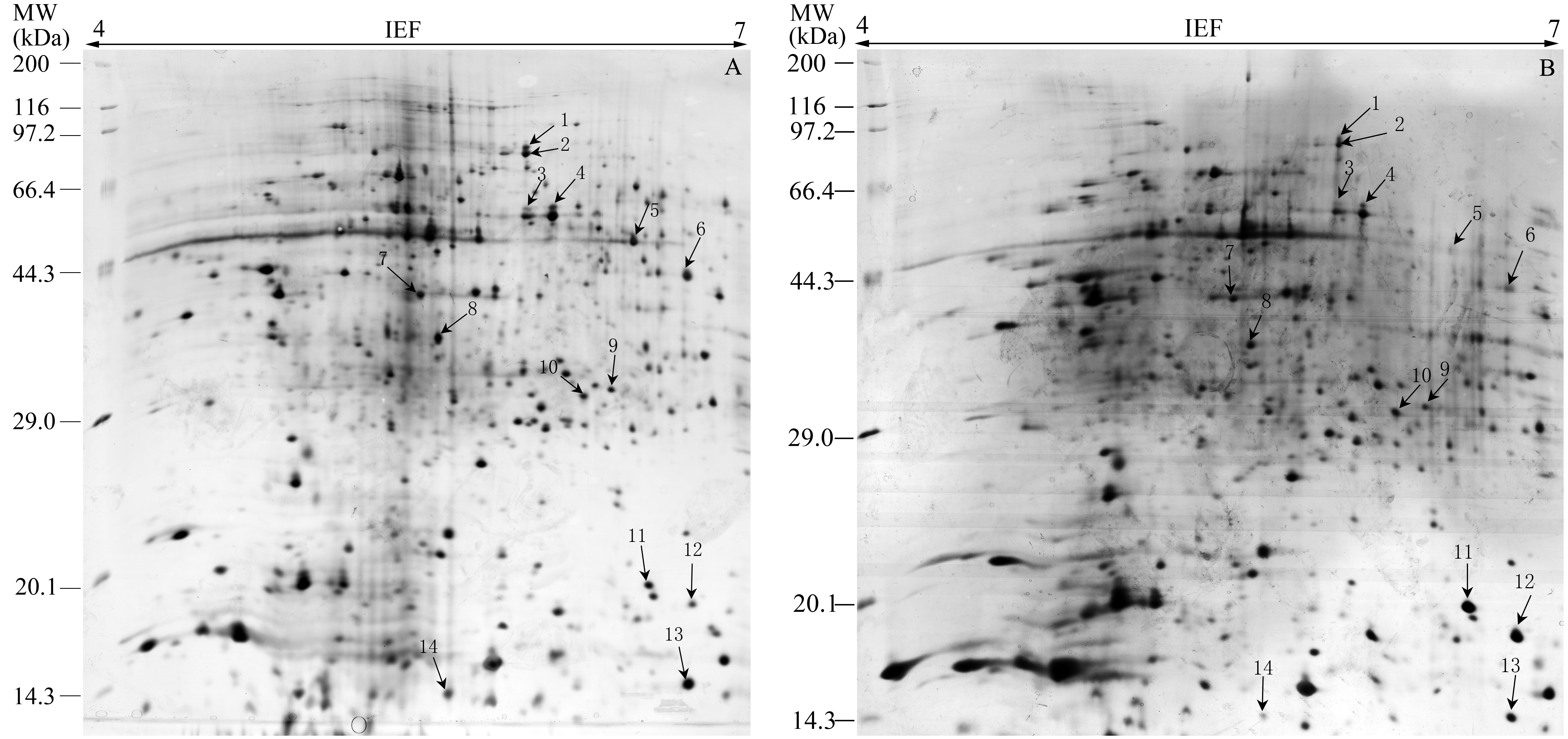
**Figure S1** Representative gel images of proteins from L4 larval stage N2 worms intoxicated by Cry6Aa2 toxin (**A**) and worms without exposure to toxin (**B**). Proteins were separated in the first dimension on an IPG strip of pH4-7, and in the second dimension on a 12% SDS-PAGE gel, followed by Coomassie Brilliant Blue G-250. An equal amount of total protein extract (200 µg) was loaded in each gel.
